# Transcriptional profiling of macaque microglia reveals an evolutionary preserved gene expression program

**DOI:** 10.1016/j.bbih.2021.100265

**Published:** 2021-05-07

**Authors:** M.L. Dubbelaar, C. Misrielal, J.J. Bajramovic, S.M. Burm, E.A. Zuiderwijk-Sick, N. Brouwer, C. Grit, S.M. Kooistra, S.M.O. Shinjo, S.K.N. Marie, H.W.G.M. Boddeke, B.J.L. Eggen

**Affiliations:** a Department of Biomedical Sciences of Cells & Systems, Section Molecular Neurobiology, University of Groningen, University Medical Center Groningen, Groningen, the Netherlands; b Unit Alternatives, Biomedical Primate Research Center, Rijswijk, the Netherlands; c Department of Neurology, Laboratory of Molecular and Cellular Biology (LIM15), Faculdade de Medicina FMUSP, Universidade de São Paulo, São Paulo, Brazil; d Center for Healthy Ageing, Department of Cellular and Molecular Medicine, University of Copenhagen, Blegdamsvej 3B, 2200, Copenhagen, Denmark

**Keywords:** Microglia, Transcriptomics, Macaque, Mouse, Human, Zebrafish

## Abstract

Microglia are tissue-resident macrophages of the central nervous system (CNS), and important for CNS development and homeostasis. In the adult CNS, microglia monitor environmental changes and react to tissue damage, cellular debris, and pathogens. Here, we present a gene expression profile of purified microglia isolated from the rhesus macaque, a non-human primate, that consists of 666 transcripts. The macaque microglia transcriptome was intersected with the transcriptional programs of microglia from mouse, zebrafish, and human CNS tissues, to determine (dis)similarities. This revealed an extensive overlap of 342 genes between the transcriptional profile of macaque and human microglia, and showed that the gene expression profile of zebrafish is most distant when compared to other species. Furthermore, an evolutionair core based on the overlapping gene expression signature from all four species was identified. This study presents a macaque microglia transcriptomics profile, and identifies a gene expression program in microglia that is preserved across species, underscoring their CNS-tailored tissue macrophage functions as innate immune cells with CNS-surveilling properties.

## 1 Introduction

Microglia, the macrophages of the central nervous system (CNS), account for approximately 10% of the cells in the CNS (Lawson et al., 1990; Mittelbronn et al., 2001), and are important for the maintenance of tissue homeostasis. Microglia scan their surrounding microenvironment and upon homeostatic disturbances or infection, they become activated, secrete inflammatory cytokines, phagocytose cellular debris, and support tissue remodeling (Kettenmann et al., 2011; Holtman et al., 2015). In case of an inflammatory insult or tissue damage, microglia can transition into a range of activation states, which are often accompanied by changes in both morphology and gene expression (Nimmerjahn et al., 2005; Block et al., 2007; Hickman et al., 2008; Gomes-Leal, 2012; Hickman et al., 2013).

Genome-wide gene expression profiling of mouse microglia revealed a gene expression program distinct from other CNS cell types and other tissue-resident macrophages (Hickman et al., 2013; Galatro, Holtman, et al., 2017). These microglia-specific genes reflect the functional properties of microglia like phagocytic activity and innate immunity but also CNS-tailored functions like neuronal support (Sierra et al., 2010; Cunningham et al., 2013) and synaptic pruning (Wake et al., 2009; Paolicelli et al., 2011). Expression profiling studies of microglia primarily have been performed in humans and rodents. Transcriptome analysis of human microglia indicated an extensive overlap with the gene expression profile of mouse microglia (Galatro, Holtman, et al., 2017; Gosselin et al., 2017). However, there are also differences between microglia from humans and commonly used animal models, especially with regards to aging-associated changes in gene expression (Galatro, Holtman, et al., 2017; Gosselin et al., 2017). In mice, genes encoding proteins involved in the recognition of endogenous ligands are downregulated while genes required for microbe recognition and host defense were more abundantly expressed (Hickman et al., 2013). In humans, changes were observed in genes involved in cell adhesion, axonal guidance, and actin (dis)assembly (Galatro, Holtman, et al., 2017). Recently, an extensive study reported a transcriptional profile of microglia from species spanning 450 million years of evolution (Geirsdottir et al., 2019). Microglia in different species are considered to be quite similar and displayed relatively homogenous homeostatic states; but human microglia were reported to be most heterogeneous. Comparison of rodents and primate microglia revealed gene expression differences (Geirsdottir et al., 2019).

Primates, like the rhesus macaque (*Macaca mulatta),* are phylogenetically closely related to humans (Evogenao, no date) and of value (Phillips et al., 2014). A detailed characterization of (dis)similarities between microglia from primate and other mammals or eukaryotes might lead to a better understanding of human biology and the vulnerability of the human CNS to pathology (Medical Research Council (MRC) and Wellcome Trust, no date).

In this study, microglia gene expression profiles isolated from healthy outbred rhesus macaques were generated and compared to the human microglia (*Homo sapiens)* transcriptome (Galatro, Holtman, et al., 2017). Microglia gene expression profiles from zebrafish (*Danio rerio)* (Oosterhof et al., 2017) and mouse (*Mus musculus)* (Hickman et al., 2013) were used in a meta-analysis to identify a core microglia profile shared by these four species.

## 2 Methods

### 2.1 Animals

Data reported in this study was acquired from the subcortical frontal brain region of rhesus macaques (*Macaca mulatta*) (Zuiderwijk-Sick et al., 2007). These animals were outbred and housed in an open environment. Brain tissues of donor monkeys were collected during necropsy from the out bred colony that is maintained at the Biomedical Primate Research Centre in Rijswijk, The Netherlands. This facility complies with the Dutch law on animal experiments. Detailed information on the animals that were included in our study, such as age and sex, are listed in Suppl. Table 1.

**Microglia isolation.** Microglia were isolated as previously described (Galatro, Vainchtein, et al., 2017). In brief, the subcortical frontal brain regions from rhesus macaque were collected in ice-cold HBSS (Lonza, Switzerland) supplemented with 15 ​mM HEPES (Lonza) and 0,6% (wt/vol) glucose (Sigma-Aldrich, USA). Brain tissues were minced and mechanically dissociated using a glass tissue homogenizer followed by a filtering step using a 300 ​μm sieve and followed by a 106 ​μm sieve to obtain a single-cell suspension. Cells were pelleted by centrifugation at 220×*g* for 10 ​min (acc: 9, brake 9, 4 ​°C). The pellet was resuspended in a solution of 22% Percoll (GE Healthcare, UK), 40 ​mM NaCl and 77% myelin gradient buffer (5,6 ​mM NaH_2_PO_4_, 20 ​mM Na_2_HPO_4_, 140 ​mM NaCl, 5,4 ​mM KCl, 11 ​mM glucose, pH 7.4). A layer of PBS was added on top and this gradient was centrifuged at 950×*g* for 20 ​min (acc: 4, brake: 0, 4 ​°C) to separate the cells from the myelin. The myelin layer and remaining supernatant were carefully removed and the pellet was resuspended in a solution of 60% Percoll, which was overlaid with respectively 30% Percoll and PBS, and centrifuged at 800×*g* for 25 ​min (acc: 4, brake 0, 4 ​°C). The cell layer at the 60–30% Percoll interface was collected with a pre-wetted Pasteur pipette, washed and cells were pelleted by centrifugation at 600×*g* for 10 ​min (acc: 9, brake: 9, 4 ​°C). The final pellet was resuspended in HBSS without phenol red (Lonza) supplemented with 15 ​mM HEPES and 0.6% glucose.

**Staining procedure and fluorescence activated cell sorting (FACS).** Fc receptors were blocked with human Fc receptor binding inhibitor (eBioscience, 14-9161-73, USA) for 10 ​min on ice. For sorting, cells were incubated for 20 ​min on ice with anti-human CD11B-PE (BioLegend, 301306, USA) or together with anti-non-human primate CD45-FITC (Miltenyi Biotech, 130-091-898,USA) and subsequently washed with HBSS without phenol red. The cells were passed through a 35-μm nylon mesh (BD Biosciences, Switzerland), collected in round-bottom tubes (Corning, USA), and sorted with either MoFlow-XDP or MoFlo-Astrios (Beckman Coulter). Viable microglia were sorted based on either CD11B^high^ or CD11B^high^ CD45^int^ expression and exclusion for DAPI, and collected in Medium A (Qiagen, Germany). Sorted cells were centrifuged at 5000×*g* for 10 ​min and cell pellets were lysed in RLT-Plus buffer (Qiagen) for RNA extraction. After the release of an anti-non-human primate CD45 antibody we sorted CD11B^high^/CD45^int^ microglia. Ultimately, this led to the generation of two macaque cohorts: CD11B^high^ and CD11B^high^/CD45^int^ population.

### 2.2 RNA isolation, library preparation and sequencing

**CD11B**^**high**^**population.** Total RNA from FACS-sorted microglia cells was extracted using an RNeasy Plus Micro Kit (Qiagen, 74034) and from total tissue using RNeasy Lipid Tissue Mini Kit (Qiagen, 74804), according to the manufacturer's protocol. RNA quality and sequencing were similar to the procedures of human samples (Galatro, Holtman, et al., 2017). In short, the SMARTer Stranded Total RNA-Seq Kit – Pico Input Mammalian (Takara Bio USA, Mountain View, CA, USA). cDNA libraries were prepared for 25 macaque microglia samples starting with 4 ​ng of total RNA. rRNA fragments were captured and degraded with RiboGone probes and enzyme. The remaining RNAs were fragmented by heat in the presence of divalent cations. The transcript for the first-strand cDNA was prepared with reverse transcriptase and random primers. The second-strand cDNA was ligated with adaptors and an index of unique sequence were added to cDNA fragments. The remaining library fragments were enriched by PCR. Final libraries were quantified by Kapa Sybr Green qPCR Kit (Roche Diagnostics, Mannheim, Germany) and the median size of the libraries determined by TapeStation 2200 (Agilent Technologies, Santa Clara, CA, USA), using the High Sensitivity D1000 ScreenTape assay. Libraries were subjected to 2 ​× ​75 bp paired-end sequencing in a NextSeq 500 system (Illumina, San Diego, CA, USA).

**CD11B**^**high**^**/CD45**^**int**^**population.** Total RNA from FACS-sorted microglia cells were extracted using an RNeasy Plus Micro Kit (Qiagen, 74034), according to the manufacturer's protocol. RNA quantity and quality was measured using the RNA 6000 Pico Kit (Agilent Technologies, USA) or Experion RNA HighSens Analysis Kit (Bio-Rad, USA). Based on RNA quality, 23 samples with a RIN value between 5.0 and 9.3 were selected for further processing. To reduce ribosomal rRNA reads in RNA sequencing libraries Ploy(A) selection (Bioo Scientific, #NOVA-512979, USA) was carried out according to the manufacturer's protocol, using 40 ​ng total RNA. Libraries were constructed from this Poly(a) Bead selected RA using the NEXTflex™ Rapid Directional qRNA-Seq Kit (Bioo Scientific, #NOVA-5130-01D, USA). Libraries were quantified using the 2100 Bioanalyzer and High Sensitivity dsDNA kit (Agilent). Subsequently, individual libraries were pooled in equimolar ratios and randomly distributed over two sequencing pools (11 and 12 microglia samples per pool). Both pools, including PhiX (5%) as an internal control, were sequenced as a 75bp paired-end, dual index run on a NextSeq 500 system (Illumina) at the sequencing facility in the UMCG, Groningen.

### 2.3 Alignment of *Rhesus macaque* samples

**Total tissue and CD11B**^**high**^**population.** The alignment procedure of these samples was similar to the procedures previously described (Galatro, Holtman, et al., 2017). Sequence quality was determined with FastQC (Andrews, 2015). Trimming of low-quality reads and adapters was performed with bbduk of the BBTools suite (Bushnell, no date). Fastq files were then aligned with SUBREAD (version 1.5.1). After alignment, the sequences of the samples were quantified with featureCounts (Liao et al., 2014) (version 1.6.2).

**CD11B**^**high**^**/CD45**^**int**^**population.** In the NEXTflex alignment procedure, the first 9 base pairs with the cell barcode were removed using fastx_trimmer (Hannon Lab, no date) (version 0.0.14). If the 9th base pair was a thymine, the first 10 base pairs were removed. These cell barcodes were saved in a separate file that was used for demultiplexing. Fastq files were aligned to the ensembl macaque genome (Mmul_8.0.1.92) with HISAT2 (Kim et al., 2015) (version 2.1.0, default parameters). Data was quantified with featurecounts (Liao et al., 2014) (version 1.6.2) and further processed with the NEXTflex demultiplexing script (Bioo scientific, 2014) to correct for counts that were obtained through technical variation. The outcome of the demultiplexing step was used for further analysis.

**Analysis of macaque RNA-Seq data.** Data was loaded into R and transformed into a DESeq2 object (Love et al., 2014). Not expressed genes were filtered out before differential gene expression analysis. Genes with an FDR < 0.001 and a logFC > 3 were regarded as differentially expressed. These cut-offs were applied to the differential expression analysis of all species.

**Calculation delta percentile** Quantified transcriptomic reads in microglia and total tissue samples were normalized to TPM values, and used to calculate a percentile value. The mean values of all samples in a cohort were obtained, that were used to compute an empirican distribution that ranges from 0 to 100). Similarities in gene expression between the two microglia cohorts were identified by calculating the Δ percentile using the following formula: abs (distribution condition 1 - distribution condition 2). A small Δ percentile indicates similarity in expression level.

### 2.4 Macaque microglia profile

Percentile values in each cohort were calculated, and genes in the top 10 percentile were considered to be most abundantly expressed. These three lists were then compared to determine (i) the highest expressed genes in both microglia cohorts, and (ii) the genes most abundantly expressed in the CD11B^high^/CD45^int^ population. Furthermore, genes that had a Δ percentile < 20 (previously described) were retained. Additionally, an expression distribution (in %) of each gene over total tissue, CD11B^high^ and CD11B^high^/CD45^int^ was calculated and visualized in a tertiary plot. Based on these values, a 60% percentage cut off was included to obtain a maqacue cluster that was microglia specific.

**Gene ontology.** Gene ontology (GO) term enrichment was performed with a combination of the clusterProfiler and enrichplot libraries (Yu et al., 2012). Given an input list with genes, gene functions with a p-value < 0.05 and q-value < 0.1 were kept for further analysis. This led to the identification of various GO annotations that were reduced in numbers through the combination of GO's with a similar gene functions. A vector of children of the root node (GO:0008150) was generated with the get_child_nodes function of the GOfuncR package (Grote, 2019). Children with a distance of less than 3 were kept as a reference for the other GO annotations. For each GO that was found per organism, the parents (and grandparent) nodes were obtained and returned as a matrix. The parent that matched with a reference GO was used for re-annotation and the corresponding gene numbers were saved in an adjusted matrix that consisted of the original GO identifier, chosen parent GO identifier, and parent GO description. The second part of this adjustment was to obtain genes of the original GO terms and to count them to determine the number of unique genes that where present in the re-annotated GO terms. This information was used as input for the circos plots.

**Gene lists of other species.** The microglia profile from zebrafish (Oosterhof et al., 2017), mouse (Hickman et al., 2013) and human (Galatro, Holtman, et al., 2017) were obtained from GEO (Edgar et al., 2002; Wang, Lachmann and Ma'ayan, 2019). The isolation method of the mouse and human data used the same sorting strategy as the macaque samples (CD11b/CD45). However, transgenic zebrafish expressing macrophage-expressed gene 1 (mpeg1)-GFP were used in the study Oosterhof et al. (2017) for the isolation of microglia. Mouse microglia transcriptomes from Hickman et al. (2013) were used, consisting of 2 microglia and 2 total tissue samples (5 months). These samples were processed with the aligning pipeline of BRAIN-SAT (Dubbelaar et al., no date), loaded in R, and analyzed with DESeq2 (Love et al., 2014). Genes that were more highly expressed in microglia samples (FDR ​< ​0.001 and logFC ​> ​3) were compared with different organisms to retain the information of the original study. Genes expressed in microglia (FDR ​< ​0.001 and logFC ​> ​3) from zebrafish (Oosterhof et al., 2017) and human (Galatro, Holtman, et al., 2017) were obtained similary.

**The interconnectivity observation of the lenient subset and evolutionary preserved core.** The lenient subset consists of genes that were present in three of the four species, whereas the evolutionary preserved core only includes genes that were overlapping among all species. Both gene subsets were uploaded in StringDB (Szklarczyk et al., 2019) where the gene symbols were used to calculate the protein-protein interaction, which was then loaded in Cytoscape (Shannon, 2003). A distinction was made between the evolutionary preserved core (blue) and lenient core (grey). The width of the connective lines reflect the confidence of two proteins interacting.

## 3 Results

### 3.1 Acute isolation of microglia from rhesus macaque CNS using CD11B

Rhesus macaque brain samples were collected during necropsy. From these samples, microglia were collected from the subcortical frontal brain regions by mechanical dissociation and viable cells were isolated by fluorescence-activated cell sorting (FACS) using CD11B^high^ as a microglia marker (referred to as a CD11B^high^ population). Information regarding age and sex of the macaque samples are provided in Suppl. Table 1. RNA-sequencing was performed on 14 microglial and 5 unsorted tissue samples that were compared to identify differentially expressed genes.

**Fig. 1:**
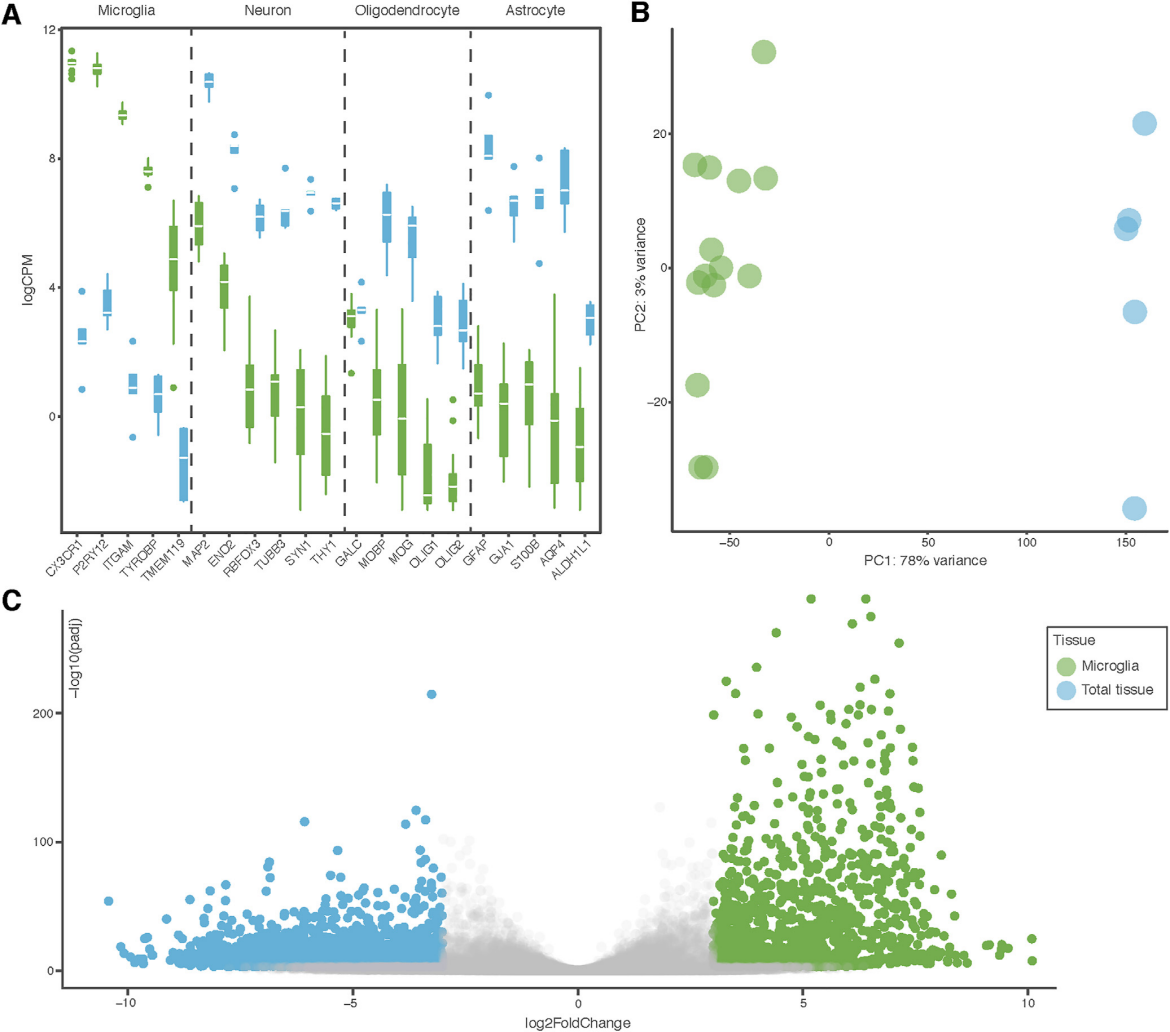
**Comparison of macaque CD11B**^**high**^**microglia and total brain tissue gene expression profiles.** (A) RNA-Seq expression of *ex-vivo* isolated microglia (n ​= ​14) compared to brain tissue samples (n ​= ​5) on known microglial, neuronal, astrocytic, and oligodendrocytes markers. The boxes contain the interquartile range, white lines indicate the median (B) Principal component analysis of microglial (green) and unsorted total brain tissue (blue) samples. Each dot represents an individual sample. (C) Volcano plot depicting genes differentially expressed between microglia and total tissue samples (logFC ​> ​3 and p-adjusted value ​< ​0.001). ∗∗p ​< ​0.01. (For interpretation of the references to color in this figure legend, the reader is referred to the Web version of this article.)

To conform the purity of the CD11B^high^ microglia samples, the expression level of CNS cell-type-specific genes was assessed (Fig. 1A). The expression levels of known microglial genes *CX3CR1, P2RY12, ITGAM, TYROBP,* and *TMEM119* was significantly higher in the CD11B^high^ microglia samples (depicted in green) compared to the total tissue samples (depicted in blue). In addition, known marker genes for neurons, astrocytes, and oligodendrocytes were more abundantly expressed in total tissue samples (Fig. 1A).

**Suppl. Fig. 1:**
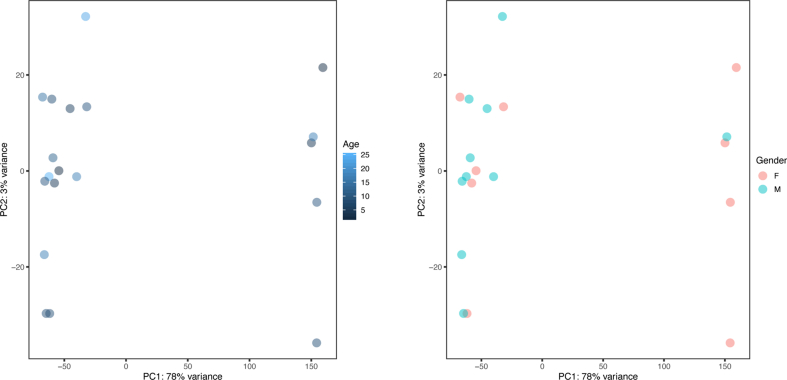
Comparison of age and sex of the macaque microglia samples. Principal component analysis of RNA-seq samples indicate no segregation based on (A) age or (B) sex. Each dot represents an individual sample.

Principal component analysis (PCA) was performed to determine variance among CD11B^high^ microglia and total tissue samples. The gene expression profiles of CD11B^high^ microglia and total tissue samples were very different, these samples segregated in PC1, which explained 78% of the variance (Fig. 1B). PCA of only CD11B^high^ microglia samples with age and sex as variables revealed no obvious segregation based on these parameters (Suppl. Fig. 1).

A differentially expression analysis was performed to reveal gene expression differences between microglia and total tissue samples. In total, 1261 genes were higher expressed in microglia compared to total tissue samples (log fold change (FC) ​> ​3, adjusted P ​< ​0.001; Fig. 1C). GO term enrichment was performed to determine functional properties associated with these microglia-enriched genes. Many significantly enriched terms were associated with the innate immune functions of microglia, such as “innate response”, “cell activation”, “cytokine production” and “activation of immune response”. Genes highly expressed in microglia (log FC ​> ​5), included several well-known microglia markers such as *TYROBP, AIF1 (IBA1), CD52, GPR84, SOX4, CABLES1,* and *ITGAM (CD11B)* (Zrzavy et al., 2017). In addition, genes that are viewed as markers of homeostatic microglia such as*, P2RY12*and *GPR34* were also highly expressed (Zrzavy et al., 2017).

### 3.2 Identification of a macaque microglial profile based on surface expression of both CD11B and CD45

As CD11B is not exclusively expressed by microglia but also by other myeloid cells, it cannot be excluded that the CD11B^high^ cell population also contained non-microglia cells. We implemented an additional cohort with the antibody for *Macaca mulatta* CD45 when it became available. Furthermore, to confirm our previous data and establish a more specific macaque microglia profile, we FACS isolated microglia based on CD11B^high^/CD45^int^ expression from 23 macaque samples.

**Fig. 2:**
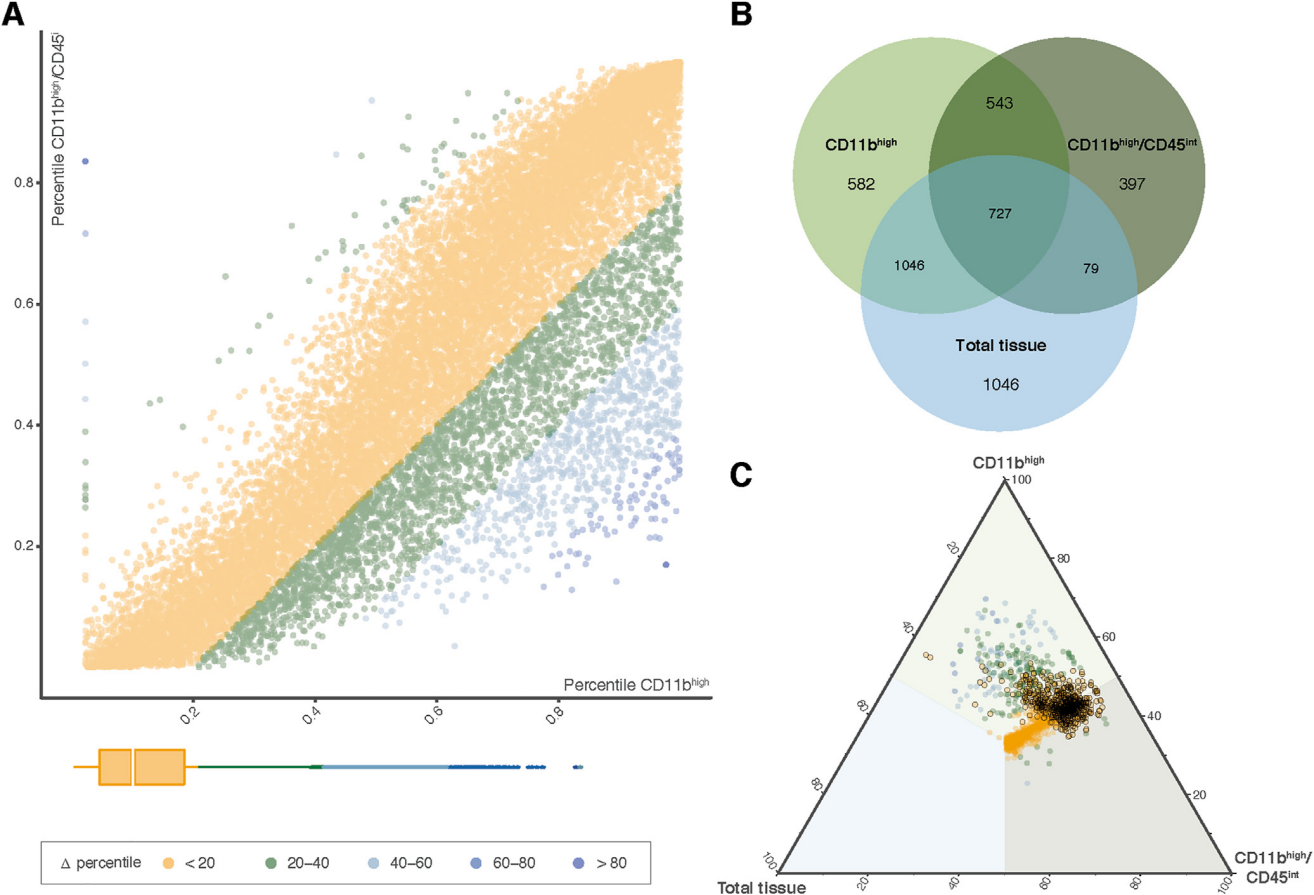
**Comparison of the CD11B**^**high**^**and C11B**^**high**^**/CD45**^**int**^**macaque microglia profiles.** (A) Dot plot depicting the delta percentile of CD11B and CD11B/CD45 microglia for individual genes. The distribution of all detected macaque genes over the Δ percentile intervals is shown in the box plot. (B) Venn diagram with the number of highest expressed (percentile 0–10) genes for the indicated conditions. (C) Triangle plot depicting macaque microglia genes (represented by dots). The axes range from 0 to 100 percent, indicating the abundance of expression of a gene in each condition. The colors of the dots indicate Δ percentile ranges. Dots with a black line are the macaque microglia profile genes. (For interpretation of the references to color in this figure legend, the reader is referred to the Web version of this article.)

We were unable to directly compare the gene expression profiles of the CD11B^high^ and CD11B^high^/CD45^in^ samples due to differences in sample processing. The similarity in gene expression levels between the CD11B^high^ and CD11B^high^/CD45^int^ microglia samples was determined using a Δ percentile approach. Genes were ranked based on their absolute expression level in each data set. Their respective percentiles in each data set were subtracted to obtain the change in percentile (Δ percentile) (Fig. 2A). The median Δ percentile was 9.53, indicating that many genes were comparably expressed between the CD11B^high^ and CD11B^high^/CD45^int^ samples.

**Fig. 3:**
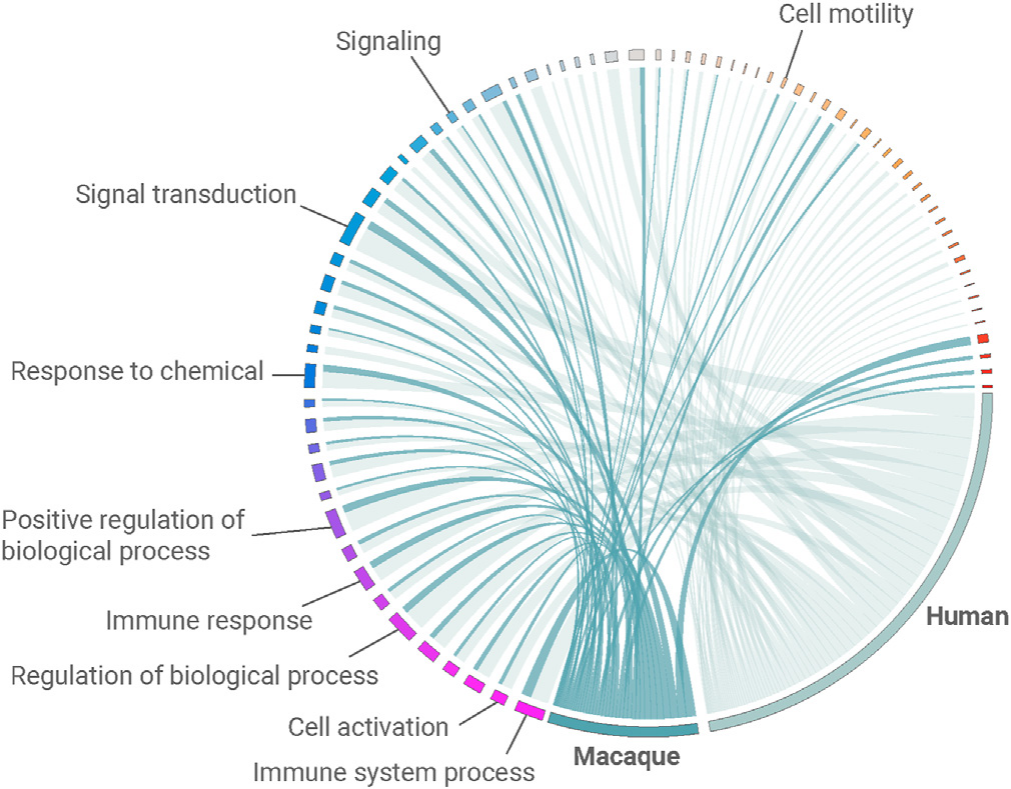
**GOs associated with human and macaque microglia.** Circos plot consisting of gene ontology parents were each gene ontology is represented by a color that ranges from purple to red. The size of the gene ontology block represents the number of annotated genes. Human and macaque gene ontology are indicated in grey and blue, respectively. The ribbons indicate the species (color) and relative contribution (width) to each gene ontology term. (For interpretation of the references to color in this figure legend, the reader is referred to the Web version of this article.)

A quantitative expression analysis approach was used to identify highly expressed genes in the CD11B^high^/CD45^int^ population. Genes were ranked using the percentiles in all three sample groups (total tissue, CD11B^high^ and CD11B^high^/CD45^int^), genes in the top 10 percentile were considered to be highly expressed. Highly expressed genes in total tissue were removed from the microglia gene list to generate a “microglia unique” gene set (Fig. 2B). This approach resulted in 543 genes that were shared in both populations and 397 genes that were only highly expressed in the CD11B^high^/CD45^int^ samples. GO term enrichment was performed on these 940 genes that revealed functions as “activation of the immune response”, “cytokine production” and “innate response”. Highly expressed genes included marker genes like: *IL1B*, *ITGAM (CD11B), TMEM119*, *TREM2,* and *VSIR.*

**Suppl. Fig. 2:**
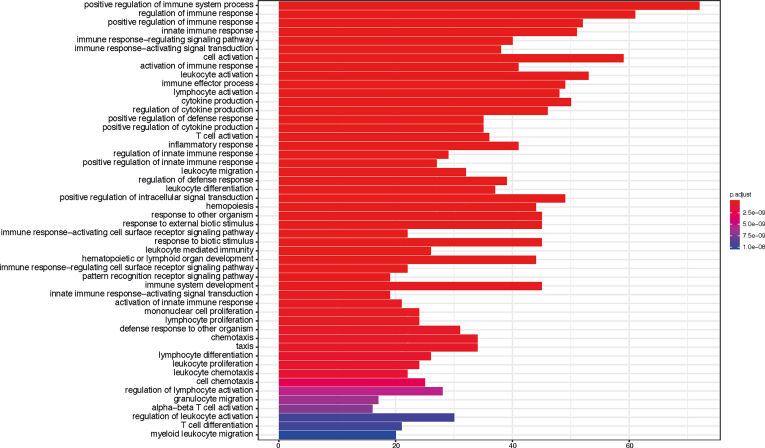
Gene annotations of the macaque microglia profile. Gene terms associated with the 666 genes of the macaque microglia profile. The number of genes in each GO is represented by the length of the bar, the color indicates significance significance, ranging from blue (1.0 ​E−10) to red (2.5 E−9).

A macaque microglia gene expression profile was generated by combining the genes that were more abundantly expressed (FDR ​< ​0.001 and logFC ​> ​3) in microglia compared to total tissue samples (Fig. 1C) with the 940 genes that were most abundantly expressed in microglia based on quantitative expression levels (Fig. 2B, Suppl. Table 2). Percentages of the microglia-enriched genes of all three sample groups (total tissue, CD11B^high^ and CD11B^high^/CD45^int^), are depicted in a triangle plot to determine if the identified genes are microglia specific (Fig. 2C). Genes that were more abundantly expressed in microglia based on differential gene expression analysis with total brain samples were combined with the highest expressed genes from two populations: i) the highest expressed genes in both microglia cohorts complemented with ii) the genes most abundantly expressed in the CD11B^high^/CD45^int^ population. Only genes with a Δ percentile < 20 were retained. In addition, a percentage distribution of each gene over total tissue, CD11B^high^ and CD11B^high^/CD45^int^ was calculated. Furthermore, the gene expression needed to be higher in microglia then in total tissue (>60%) before it was considered as a microglia specific gene. Genes with a Δ percentile < 20 and a microglia percentage > 60% were included in the macaque core microglia profile, which consisted of 666 genes, and is indicated by dots surrounded with a black line (Fig. 2C, Suppl. Fig. 2).

### 3.3 Identification of a primate microglial gene profile

**Suppl. Fig. 3:**
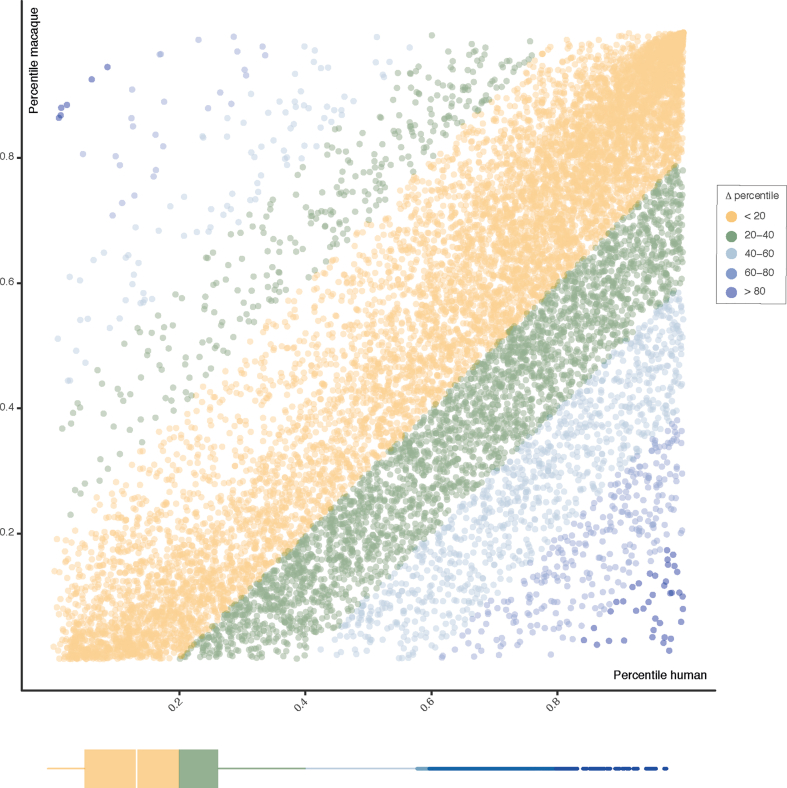
Comparison of the macaque and human microglia profiles. Dotplot depicting the delta percentile of the macaque and human microglia for individual genes. The distribution of all detected macaque genes is indicated with a Δ percentile interval, this information is shown in the box plot.

To identify a primate microglia gene expression profile, we compared the macaque microglial expression profile obtained from the subcortical frontal brain region to a published human transcriptome of parietal cortical microglia (Galatro, Holtman, et al., 2017). The similarity between these two transcriptomes was determined by calculating Δ percentiles. The expression levels of many microglia genes were similar between macaques and humans, indicated by a median Δ percentile of 13.89 (Suppl. Table 3; Suppl. Fig. 3). Overlaying the transcriptional profiles of human and macaque microglia revealed 342 genes that were expressed in both species. GO terms associated with these genes are related to typical microglia functions such as: “phagocytosis”, “leukocyte cell-cell adhesion” and “pattern recognition receptor signaling pathway”, and included several known microglial genes such as *ITGAM, GPR56,* and *TMEM119*.

Most of the non-overlapping genes of the macaque microglia profile are related to functions in: “antigen processing and presentation”, “positive regulation of the immune response” and “T-cell activation”. These functions are not unique for macaque and this lack in overlap was caused by differences in annotations of antigens in the major histocompatibility complex (MHC). Furthermore, of the 324 non-overlapping genes, 112 transcripts did not have a gene symbol, are open reading frames (ORFs), or were annotated starting with ‘MAMU’ or ‘LOC’, suggesting the functionality of these transcripts is not yet known.

To investigate similarities in gene functions between both species, genes of both microglia profiles were annotated to GO terms (Suppl. Table 4). For visualization purposes, GOs with a common parent were combined (Fig. 3). Several immune-related processes were shared between macaque and human microglia, such as “cell activation” and “immune-response”. In addition, homeostatic processes in microglia such as “cell-motility” and “regulation of biological process”, were also shared between both species. Some GOs were only associated with human or macaque microglia, however, these annotations generally consisted of a relatively low number of genes (see Suppl. Data 4). In short, we created a macaque microglia profile that showed a considerable overlap with human microglia in expression of homolous genes and associated biological functions.

### 3.4 Identification of a core microglia gene expression profile that is preserved between species

Zebrafish and mice are well established and widely used as model organisms in biomedical research (Kalueff et al., 2014; Gurumurthy and Lloyd, 2019). Although these species are evolutionary more distant to humans than macaques, it is of interest to determine the transcriptional overlap between the microglia profiles of human with macaque, mice, and zebrafish to reveal the gene expression (dis)similarities. The macaque and zebrafish microglia profiles showed an overlap of 96 genes, such as *TLR7*, *TMEM173*, and *C5AR1,* most of which were involved in immune response regulation. However, most zebrafish transcripts were non-overlapping, which could be explained by the evolutionary distance between zebrafish and macaque. When macaque and mouse microglia profiles were compared, 186 overlapping genes were detected, including known microglia genes such as *P2RY6*, *PYCARD*, *ITGAM*, and *TMEM119*. However, several genes from the zebrafish and mice microglia profiles did not overlap as these transcripts had no or different gene symbol annotations, or were unique for the respective species.

**Fig. 4:**
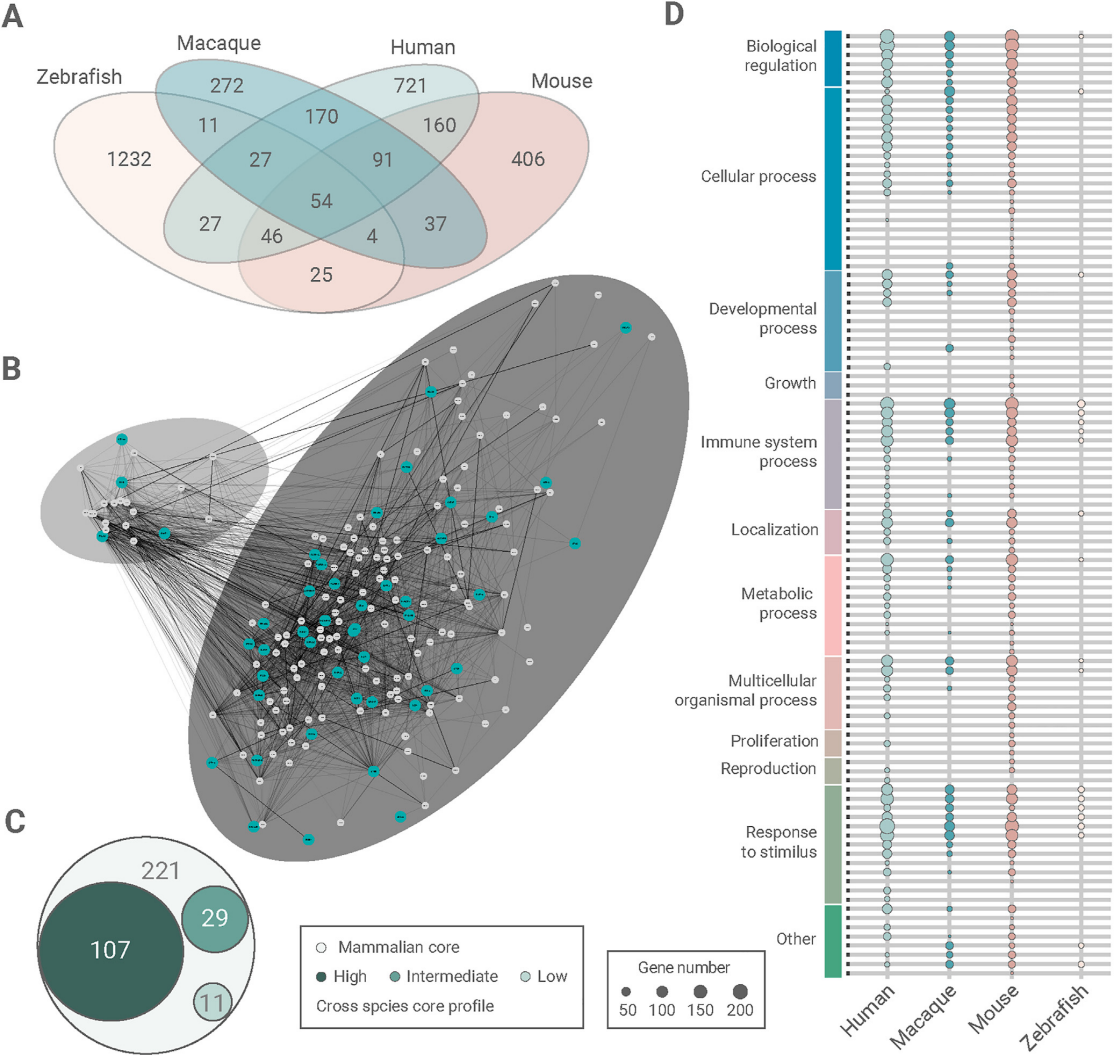
**Comparison of the macaque, human, zebrafish, and mouse microglia transcriptomes.** (A) Venn diagram depicting the overlap in genes between human (Galatro, Holtman, et al., 2017), macaque, mouse (Hickman et al., 2013), and zebrafish (Oosterhof et al., 2017) microglia. (B) Interconnectivity plot showing the 221 genes from the lenient subset, that can be divided into two clusters. The blue dots indicate genes that are present in the evolutionary preserved core and the width of the lines indicate the confidence score. Figure was generated with Cytoscape (Shannon, 2003). (C) Circos plot depicting the overlap with the high, intermediate, or low expressed genes reported in (Geirsdottir et al., 2019) with the 221 representing our lenient subset. (D) Gene functions associated with the gene ontology terms are depicted for all genes per organism. The size of the dot indicates the number of genes in the associated gene ontology term. (For interpretation of the references to color in this figure legend, the reader is referred to the Web version of this article.)

We were able to obtain a “lenient” subset of 221 genes where a gene was present in at least three of the four species. This lenient core included genes that were associated with the immune activity of microglia; “immune response-activating signal transduction”, “innate immune response” and “cytokine production”. Recently, a cross-species signature of mammals of 4714 genes was reported, divided into high, intermediate, and low expressed clusters (Geirsdottir et al., 2019). 67% of our lenient profile overlapped with this cross-species signature with a distribution over high, intermediate, and low expression clusters of 49%, 13%, and 5%, respectively (Geirsdottir et al., 2019) (Fig. 4C). Typical microglia genes were present in this overlap, such as *ALOX5AP, DOCK8, IL1B, ITGAM, PYCARD, SLA, TLR7,* and *TREM2* (Suppl. Table 5). Further exploration of the genes from the lenient subset revealed two subclusters. The smaller cluster is associated with immune-related processes, whereas the big cluster is not limited to one respective process (Fig. 4B). Furthermore, we wanted to highlight the genes that were overlapping among all species (indicated as blue dots) that is referred as the evolutionary preserved microglia core (Fig. 4A). This showed no distinction in the interconnectivity plot.

The evolutionary preserved microglia core, that consists of 54 genes, contained several established microglia transcripts, such as *TGFBR2*, *P2RY6*, *ITGAM*, and *PYCARD*. GO annotation of this preserved profile associated these genes with immune activity (Suppl. Fig. 4); “inflammatory response”, “regulation of the innate immune response” and “cytokine production”. GO annotation (Suppl. Data 6) of the different microglia profile of each species were visualized in Fig. 4D. Although the number of genes per GO varied, processes as “response to stimulus”, “regulation of biological processes”, “transport” and “cell motility” were identified in all four species. Summarizing, these findings reveal an evolutionary preserved core that was shared among all four species and a lenient subset of 221 genes. This showed that a majority of the immune-related processes in microglia were preserved during evolution.

## 4 Discussion

In this manuscript, we present a macaque microglia transcriptomic profile, which is compared to the microglia transcriptome profile of zebrafish, mouse, and human. Zebrafish and mice are often used for research purposes, however, there is a large evolutionary distance between these two species and humans. This manuscript focusses on establishing a macaque gene expression microglia profile and its comparison with microglia transcriptomes of other species.

The first aim of this study was to generate a macaque microglial transcriptomic profile. The combination of two macaque microglia cohorts, obtained using two different procedures (lab and bioinformatics), and stringent filter criteria led to the identification of a macaque microglia transcriptomic profile that is highly specific. Ultimately, this resulted in the identification of a profile of 666 genes.

An extensive overlap between the human and macaque microglia transcriptomic profiles was observed. Overall, gene expression levels were relatively similar (Suppl. Fig. 3), and 51% (342 genes) of the macaque microglia profile could also be identified in human microglia, of which several genes were previously classified as ‘human microglia specific genes’ (Galatro, Holtman, et al., 2017), and were not detected in mice. Notable examples are *FCAR* (*CD89*), which is involved in cytokine production, and many *SIGLEC* genes which are important for regulation of activation and phagocytosis (Galatro, Holtman, et al., 2017). Moreover, GO comparison led to the identification of shared gene functions that are related to the immune system (“cell adhesion”, “cell activation” and “response to bacterial components”) and “phagocytosis”. A substantial number of the 49% (324 genes) non-overlapping genes are involved in the activation of the immune response (signal transduction, antigen processing, and T-cell activation). Additionally, 17% (112 genes) of this non-overlapping gene set lacked a gene symbol, or are identified as ORF, specific locus or a macaque specific antigen. Better annotation of the macaque genome is required to determine if these genes are indeed macaque-specific or yet unidentified human homologs. Regardless, these outbred rhesus macaques, housed in an open environment, more closely reflect humans than inbred laboratory animals housed under SPF conditions.

To identify similarities between the transcriptional profiles of macaque and other species, genes in the macaque microglia profile were first compared to the zebrafish and mouse profiles, revealing an overlap of 96 and 186 genes, respectively. More genes overlapped with mice, which could be caused by a better annotation of mouse genes and transcripts. The zebrafish transcriptome remains ambiguous due to the lower number of transcriptomic analysis performed in this species. Similarities between macaque and mouse microglia include several known microglia genes and gene functions including immune response, phagocytosis, cytokine secretion, and myeloid cell differentiation. When the mouse and macaque microglia profile are compared, we can observe that the macaque microglia profile roughly consists of the same number of genes. As expected from an evolutionary point of view, the macaque microglia profile showed a higher overlap with the human microglia profile (51%), than the mouse microglia profile (43%).

We identified a lenient microglia gene subset that was preserved in at least three of the four species, and an evolutionary preserved core that consisted of genes that were detected in all species. The comparison of the lenient and evolutionary preserved core to the cross-species signature (Geirsdottir et al., 2019), revealed an overlap of respectively 147 and 25 genes, respectively, confirming that these genes are highly expressed by microglia across evolution and species. Potential differences in gene expression in microglia between different species are difficult to detect, due to confounding factors such as different isolation procedures and sample preparations used, differences in analyzed brain regions, etc.

In conclusion, we present a specific macaque microglia gene expression profile and intersecting this macaque profile with previously described transcriptomes of zebrafish, mouse and human, we identified a lenient and an evolutionary preserved core, that show the typical functions of microglia as CNS macrophages. This study provides a valuable tool that will contribute to further exploration of the macaque microglia profile, and the evolutionary preserved microglia genes in function and development.

## Declaration of competing interest

The authors declare no conflict of interest.

## Appendix A Supplementary data

Suppl. Fig. 4 Lenient subset with associated gene functions. Gene ontology terms associated with the 221 genes overlapping of the lenient subset. Results were obtained via Metascape (https://metascape.org). Suppl. Fig. 4

Suppl. Table 1 macaque sample information. The origin, sex, age, and necropsy date are provided for the macaques used in this study. Suppl. Table 1

Suppl. Table 2 Macaque microglia profile genes. List of all genes with information from the differential and quantitative gene expression analysis. Additionally, the 666 genes in the macaque microglia transcriptomic profile are annotated. Suppl. Table 2

Suppl. Table 3 Percentile values of macaque and human microglia genes. Raw data of the percentile values and Δ percentiles used for the comparison of the macaque and human microglia profile. Suppl. Table 3

Suppl. Table 4 GO's of human and macaque microglia profile. This document consists of four tabs. The first tab, consists of all the identified gene ontology terms from the genes in the human microglia profile, and the reduced gene ontology terms to common parents in the second tab. For macaque microglia profile the third tab contains the complete gene ontology annotation and the last tab contains the reduced GO's. Suppl. Table 4

Suppl. Table 5 Comparison of the core microglia profile with Geirsdottir et al. The overlap of our mammalian and evolutionary microglia profile are compared with the microglia core that was identified by Geirsdottir et al., and subdivided into a high, intermediate and low classification. Overlapping genes of our evolutionary microglia profile (including zebrafish) are found in the highest classification of Geirsdottir et al. Suppl. Table 5

Suppl. Table 6 reduced GO's of zebrafish, mouse, macaque and human. This document consists of the information regarding the GO reduction of all species. The original gene ontology id's and terms of each species are provided with the number of genes that were associated to these terms. Furthermore, a column in provided that consists of the reduced gene ontology term, which is corresponding to the names in Fig. 4D. Suppl. Table 6
