# Supplementary figures and images for: Transcriptional profiling of macaque microglia reveals an evolutionary preserved gene expression program
Source: Brain Behav Immun Health. 2021 May 7;15:100265. doi: 10.1016/j.bbih.2021.100265 (PMC8474495; doi:10.1016/j.bbih.2021.100265)

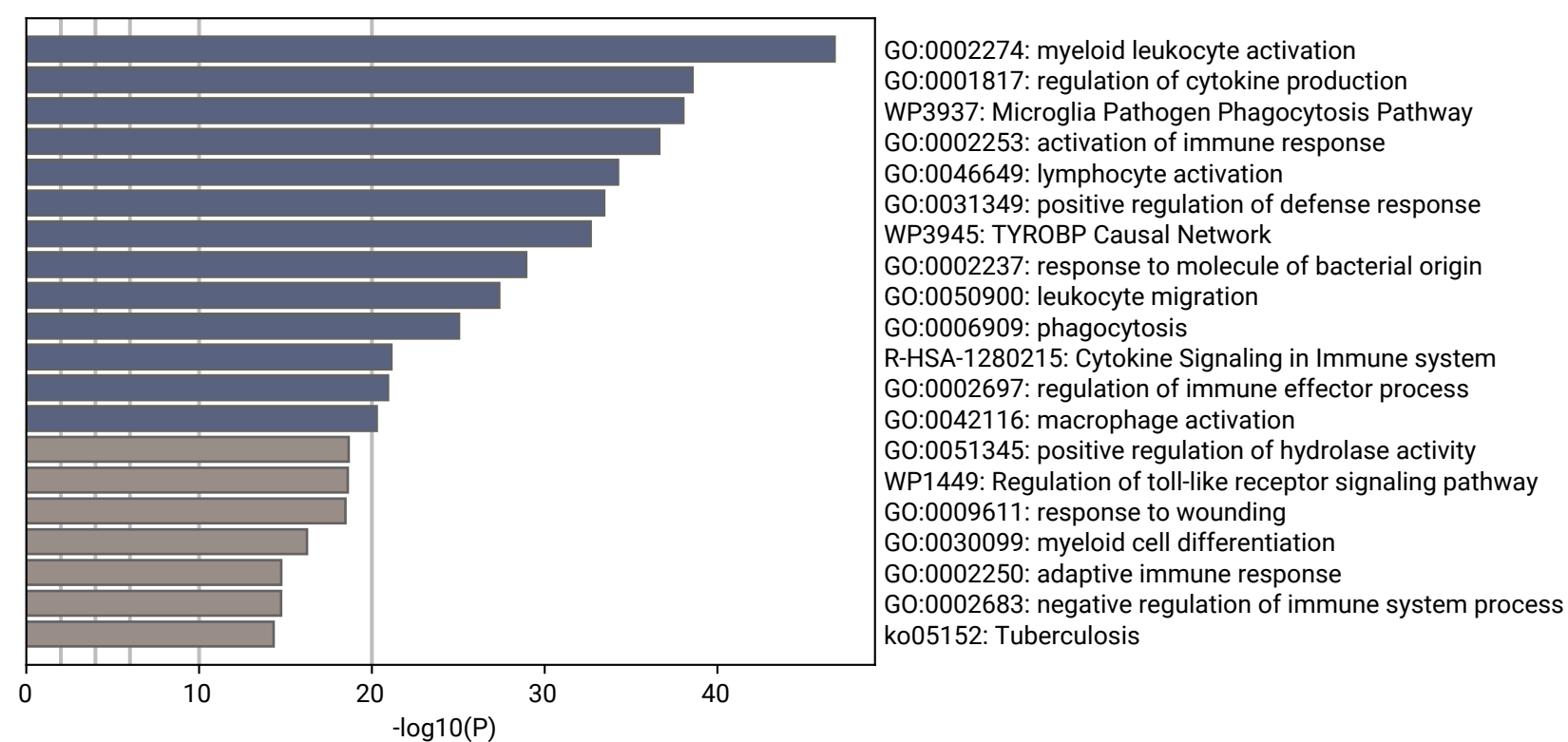

Supplement: Suppl. Fig. 4 — Lenient subset with associated gene functions. Gene ontology terms associated with the 221 genes overlapping of the lenient subset. Results were obtained via Metascape (https://metascape.org). [file mmc1.pdf]
